# Preoperative aspartate aminotransferase to albumin ratio correlates with tumor characteristics and predicts outcome of hepatocellular carcinoma patients after curative hepatectomy: a multicenter study

**DOI:** 10.1186/s12893-022-01751-4

**Published:** 2022-08-09

**Authors:** Wei Peng, Junyi Shen, Junlong Dai, Shusheng Leng, Fei Xie, Yu Zhang, Shun Ran, Xin Sun, Tianfu Wen

**Affiliations:** 1 Department of Liver Surgery & Liver Transplantation Center, West China Hospital, Sichuan University, Chengdu, 610041 Sichuan Province China; 2 Chinese Evidence-Based Medicine Center, West China Hospital, Sichuan University, Chengdu, 610041 China; 3 Department of Hepatobiliary and Pancreatic Surgery, the Affiliated Hospital of Chengdu University, Chengdu, 610072 Sichuan Province China; 4 Department of Hepatobiliary and Pancreatic Surgery, the First People’s Hospital of Neijiang City, Neijiang, 641000 Sichuan Province China; 5 Department of Hepatobiliary Surgery, Sichuan Provincial People’s Hospital, Chinese Academy of Sciences, Chengdu, 610072 Sichuan Province China; 6 Department of Hepatobiliary Surgery, Affiliated Cancer Hospital of Guizhou Medical University, Guiyang, 550000 Guizhou Province China; 7 Laboratory of Liver Transplantation, Frontiers Science Center for Disease-Related Molecular Network, West China Hospital, Sichuan University, Chengdu, 610041 Sichuan Province China

**Keywords:** Aminotransferase to albumin ratio, Hepatocellular carcinoma, Prognostic classification, Tumor characteristics, Hepatectomy

## Abstract

**Aims:**

This study aimed to evaluate the clinical significance of the preoperative aminotransferase to albumin ratio (AAR) in patients with hepatocellular carcinoma (HCC) after hepatectomy.

**Methods:**

From five hospitals, a total of 991 patients with HCC admitted between December 2014 and December 2019 were included as the primary cohort and 883 patients with HCC admitted between December 2010 and December 2014 were included as the validation cohort. The X-tile software was conducted to identify the optimal cut-off value of AAR.

**Results:**

In the primary cohort, the optimal cut-off value of the AAR was defined as 0.7 and 1.6, respectively. Compared to patients with AAR 0.7–1.6, those with AAR > 1.6 showed significantly worse overall survival (OS) and RFS, whereas those with AAR < 0.7 showed significantly better OS and RFS (all p < 0.001). Pathologically, patients with AAR > 1.6 had more aggressive tumour characteristics, such as larger tumour size, higher incidence of microvascular invasion, and severe histologic activity, and higher AFP level than patients with AAR < 0.7. Consistently, the abovementioned clinical significance of AAR was confirmed in the validation cohort.

**Conclusions:**

A high AAR was significantly correlated with advanced tumours and severe hepatic inflammation, and a worse prognosis of HCC.

**Supplementary Information:**

The online version contains supplementary material available at 10.1186/s12893-022-01751-4.

## Introduction

Hepatocellular carcinoma (HCC) is the sixth most common cancer and the fourth leading cause of cancer-related deaths worldwide, with the highest incidence in southeast Asia and sub-Saharan Africa. Hepatitis B and C virus (HBV and HCV, respectively) infections are the major etiologies of HCC [1]. Surgical resection is the main curative therapy for early-stage and selected intermediate-advanced HCCs [2]. Compared to other solid tumours, HCC commonly occurs with an underlying liver disease. Therefore, well-preserved underlying liver function is a prerequisite for surgery. Serum alanine aminotransferase (ALT), aspartate aminotransferase (AST), bilirubin (TBIL) and albumin (ALB) are the most common liver chemistry parameters for liver function assessment. Hepatic enzymes such as ALT and AST are much sensitive in the presence of acute or chronic hepatic inflammation. In patients with severe liver impairment, TBIL or ALB level becomes significantly abnormal [3]. In addition, these parameters are closely related to HCC incidence and progression. In patients with HBV, elevated ALT levels commonly indicate a high incidence of HCC development. In patients undergoing resection, ALB and TBIL are involved in HCC progression [4, 5].

The Child Pugh (C-P) system, which is deriving from five conventional liver function parameters, namely, TBIL, PT, ALB, ascites, and hepatic encephalopathy, is widely used for liver function assessment. In patients undergoing hepatectomy, C-P grade A liver function is required. Thus, the liver function parameters of patients with HCC almost fall in the normal range. Because the liver function index and HCC are closely related, the role of liver chemistry in HCC patients with C-P grade A liver function is underestimated. The clinical significance of these liver function parameters remains unclear. In 2015, the albumin-bilirubin (ALBI) scoring model for the evaluation of hepatic function in patients with HCC was reported [6]. This score completely adopted two liver function indices, namely, ALB and TBIL, to assess liver function, and the model was proven to be effective and objective. The FIB-4 (fibrosis index based on four factors) score containing ALT and AST and the APRI score (Aspartate aminotransferase to platelet ratio index) containing AST have been developed for diagnosing liver fibrosis [7–9]. These risk scores have been mainly designed for assessing underlying liver disease and has shown good predictive power. These scores further grade patients with C-P grade A liver function and have a predictive role in postoperative complications [10, 11]. However, the liver function parameters alone or in combination seldom are reported in predicting HCC prognosis after hepatectomy, particularly in terms of long-term survival. Because of the critical significance of liver function parameters, we attempted to further investigate the predictive role of liver function parameters and propose a more simple and easily used score for HCC patients undergoing hepatectomy.

Among the aforementioned liver chemistry parameters, AST and ALB were reported to be associated with HCC prognosis. Abnormal AST and ALB levels were the most prominent characteristics of HCC patients with cirrhotic hepatitis. At severe hepatic injury stage, mitochondrial lesions occurred and hepatocytes were seriously damaged, which stimulates the release of a large amount of AST, resulting in a significant increase in AST. On the other hand, due to excessive consumption by tumour progression and impaired protein synthesis ability, serum ALB levels might be decreased. Moreover, AST or ALB was identified to be a prominent prognostic factor for HCC patients [12]. Therefore, both these indices might better reflect hepatic inflammation and tumour status. In the present study, we used multi-institutional data to investigate the objective measures of liver function parameters (AST and ALB) and combined them into a risk score (the AST/ALB ratio, AAR). This score could reflect hepatic inflammation and tumour characteristics and distinguish HCC prognosis. We adopted the X-tile software to identify the optimal value. This score would be validated in another group of HCC patients after hepatectomy.

## Materials and methods

### Participants

In the present study, we enrolled 991 patients with HCC who underwent hepatectomy between January 2014 and January 2019 from five hospitals. The participating institutions included the Affiliated Hospital of Chengdu University, Affiliated Cancer Hospital of Guizhou Medical University, Sichuan Provincial People’s Hospital, the First People’s Hospital of Neijiang City, and West China Hospital of Sichuan University. The inclusion criteria were (1) age ≥ 18 years; (2) C-P grade A/B liver function; (3) primary hepatectomy; and (4) pathological HCC. The exclusion criteria were (1) multiple malignancies; (2) antitumour therapy before surgery; (3) positive surgical margin; (4) main PVTT; (5) ruptured HCC; (6) lymph node involvement; and (7) incomplete follow-up information. To validate our proposed risk score, we included another 883 patients with HCC who underwent hepatectomy between January 2010 and January 2014 from West China Hospital.

This study conformed to the ethical guidelines of the 1975 Declaration of Helsinki and was approved by the Ethics Committee of West China Hospital, Sichuan University.

### Variable collection and follow-up

Demographic information, including age and sex, and clinicopathological features, including blood test, HBV infection assessment, and liver function test findings and tumour-related parameters, were prospectively collected within 1 week before surgery. Systemic inflammatory level was evaluated using the neutrophil-to-lymphocyte (NLR) and platelet-to-lymphocyte (PLR) ratio. AAR was calculated by dividing AST by ALB. Computed tomography (CT) or contrast-enhanced magnetic resonance imaging (MRI) were performed preoperatively to determine the tumour size and number. Tumour differentiation, microvascular invasion (MVI), satellite lesion, and surgical margin status were evaluated on pathology specimens. Hepatic inflammation was assessed using the G score, namely, G1, G2, G3, and G4. The degree of the G score was assessed based on the degree of necrosis and inflammation. Liver fibrosis or cirrhosis was assessed using the S score, namely, S1, S2, S3, and S4. S4 was indicative of liver cirrhosis [13]. Curative hepatectomy was defined as complete macroscopic tumour removal with a microscopically free margin. All pathological examinations were performed by experienced hepatic pathologists.

After surgery, the patients were followed up at postoperative 1 month and then at 3-month intervals in the postoperative first 1 year and every 6 months in the subsequent years. Antiviral drugs were primarily recommended for patients with HBV at the first investigation based on the current guideline. Liver ultrasonography or contrast-enhanced CT and/or MRI, along with serum tumour marker AFP, was performed during the follow-up. HCC recurrence was diagnosed as two typical imaging findings, one imaging technique showing typical features of HCC and increased alpha-fetoprotein (AFP) level of > 400 ng/mL, or confirmed by biopsy/resection. Recurrence-free survival (RFS) was defined as the time interval between the surgery and the first incidence of detectable recurrence. Overall survival (OS) was defined as the time interval between the surgery and patient death or the last follow-up. The last follow-up was performed at the end of March 2020 or until death.

### Statistical analysis

Continuous data are presented as mean (interquartile range, IQR) and were compared using Mann–Whitney U test. Age, AFP, ALT, and TBIL were graded as reported in previous studies. The cut-off value of NLR and PLR was defined as the mean value. Categorical variables are expressed as number (frequency) and were compared using χ^*2*^ or Fisher’s exact tests, as appropriate. The survival analysis was performed using the Kaplan–Meier method, and the findings were compared using log-rank test. A Cox hazard proportion model was performed to determine the independent risk factors related to RFS or OS. Variables with p < 0.05 in the univariate analysis were entered into the multivariate analysis. All statistical analyses were performed using the SPSS statistical package (version 20.0; SPSS Inc., Chicago, IL, USA) and R software version 3.6.3 (R Foundation for Statistical Computing, Vienna, Austria; www.r-project.org). p < 0.05 was considered to indicate statistical significance.

The X-tile software (version 3.6.1; Yale University, New Haven, CTA) was used to identify the optimal cut-off value of AAR to separate patients into low-risk, intermediate-risk, and high-risk groups of recurrence. This software provides a single, global assessment of every possible way of dividing a population into low-, intermediate-, and high risk of recurrence and then identifying the optimal thresholds by selecting the largest χ^2^ value [14].

## Results

### Definition of AAR

**Fig. 1:**
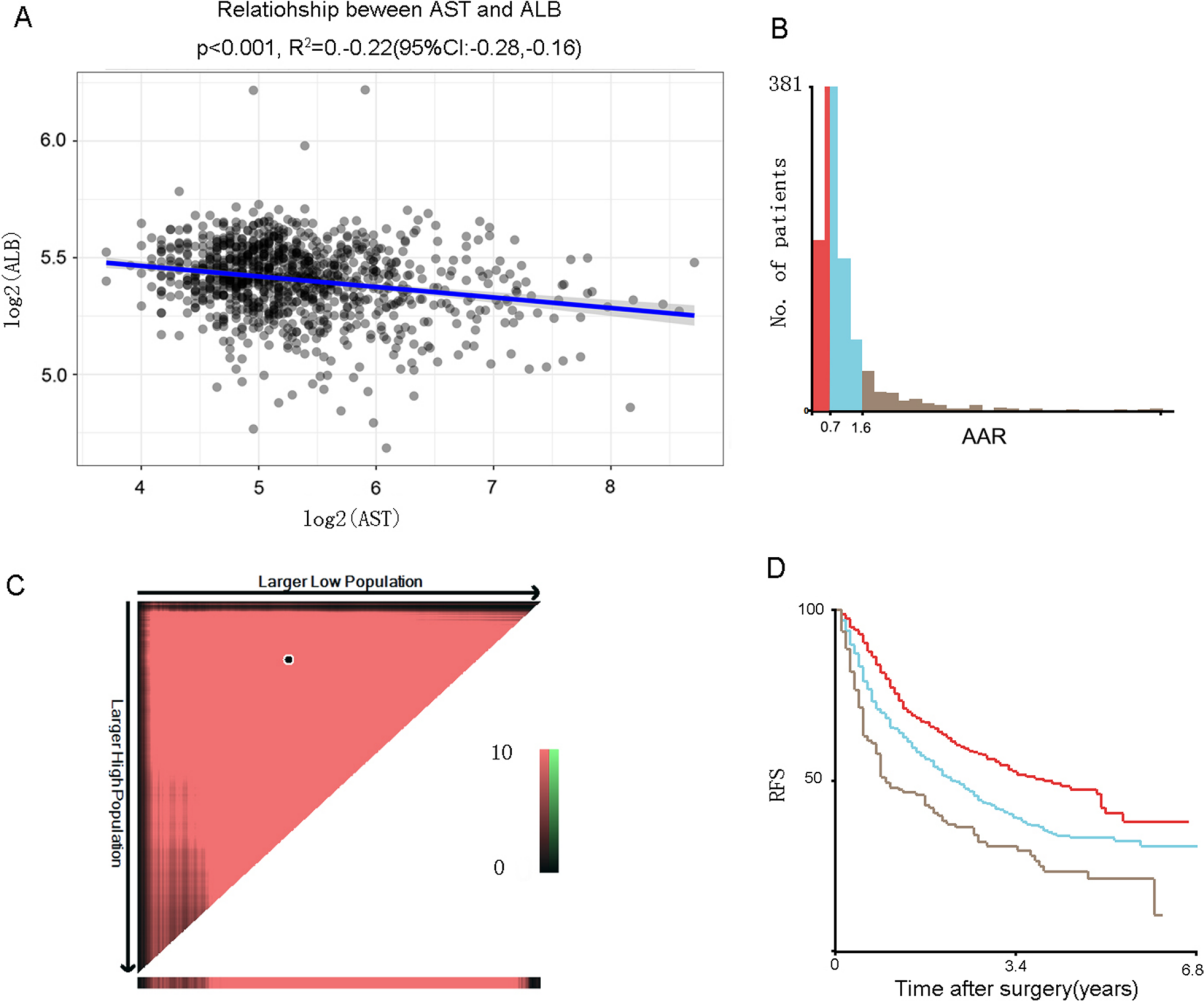
Determination of the optimal cut-off value of AAR. **A** Correlation analysis of AST and ALB (R^2^ = -0.22); **B**–**D** X-tile analysis of recurrence-free survival data for AAR, which divided AAR into the low-risk (AAR < 0.7), intermediate-risk (AAR 0.7–1.6), and high-risk (AAR > 1.6) groups. *AAR* aminotransferase to albumin ratio, *AST* aspartate aminotransferase, *ALB* albumin

To exclude collinearity, correlation analysis was performed between serum log2-transformed AST and ALB. As shown in Fig. 1A, the R^2^ value was − 0.22 (95% CI − 0.28, − 0.16), suggesting that both variables independently reflect the status of the underlying liver disease. AAR was calculated as the ratio of preoperative serum AST to serum ALB. Of the 991 patients enrolled in this study, we used X-tile plots to generate two optimal cut-off values, namely, 0.7 and 1.6, which categorises resection HCC patients into three strata with a highly different probability of RFS: low-risk (AAR < 0.7, n = 323), intermediate-risk (AAR 0.7–1.6, n = 514), and high-risk (AAR > 1.6, n = 154) (Fig. 1B–D). With AAR < 0.7 category as the reference, the hazard ratios (HRs) for AAR 0.7–1.6 and AAR > 1.6 categories were 1.833 (95% CI 1.358–2.476) and 3.688 (95% CI 2.624–5.183), respectively.

### Relationship of AAR with clinicopathologic parameters

Table 1 shows all patient demographics and clinical characteristics. There were 830 (83.8%) male patients. The majority of patients (942, 95.1%) had a history of HBV infection, and 821 patients (82.8%) had liver cirrhosis.

**Table 1:** Baseline characteristics

Variables	All	Low-risk group	Intermediate-risk group	High-risk group	p value
	n = 991	n = 323	n = 514	n = 154	
Age (> 60 years)	231 (23.3)	71 (22.0)	124 (24.1)	36 (23.4)	0.775
Gender (male, %)	830 (83.8)	273 (84.5)	429 (83.5)	128 (83.1)	0.897
Diabetes	131 (13.2)	51 (15.8)	59 (11.5)	21 (13.6)	0.198
HBsAg	942 (95.1)	300 (92.9)	492 (95.7)	150 (97.4)	0.063
HBeAg	218 (22.0)	42 (13.0)	135 (26.3)	41 (26.6)	< 0.001
HBV-DNA (> 10^3^ IU/mL)	575 (58.0)	130 (40.2)	324 (63.0)	121 (78.6)	< 0.001
Cirrhosis	821 (82.8)	263 (81.4)	432 (84.0)	126 (81.8)	0.578
Tumor size (cm)	4.5 [3.0, 7.5]	4.0 [2.8, 5.5]	5.0 [3.0, 7.5]	8.1 [4.6, 12.0]	< 0.001
Multiple tumors	89 (9.0)	25 (7.7)	52 (10.1)	12 (7.8)	0.430
MVI	227 (22.9)	43 (13.3)	133 (25.9)	51 (33.1)	< 0.001
Capsular invasion	436 (44.0)	136 (42.1)	228 (44.4)	72 (46.8)	0.615
Satellite lesion	102 (10.3)	26 (8.0)	60 (11.7)	16 (10.4)	0.244
Tumor differentiation					0.704
Well	14 (1.4)	5 (1.5)	6 (1.2)	3 (1.9)	
Moderate	534 (53.9)	183 (56.7)	271 (52.7)	80 (51.9)	
Poorly	443 (44.7)	135 (41.8)	237 (46.1)	71 (46.1)	
AFP (> 400 ng/mL)	376 (37.9)	103 (31.9)	197 (38.3)	76 (49.4)	0.001
INR	1.04 [1.00, 1.10]	1.03 [0.98, 1.08]	1.04 [1.00, 1.10]	1.07 [1.01, 1.14]	< 0.001
BCLC staging					0.900
0	64 (6.4)	30 (9.3)	29 (5.6)	5 (3.2)	
A	858 (86.6)	276 (85.4)	442 (86.0)	140 (9.0)	
B	69 (7.0)	17 (5.3)	43 (8.4)	9 (5.8)	
PLR (> 103.5)	382 (38.5)	114 (35.3)	183 (35.6)	85 (55.2)	< 0.001
NLR (> 2.6)	327 (33.0)	91 (28.2)	167 (32.5)	69 (44.8)	0.001
ALT (> 40 IU/L)	403 (40.7)	38 (11.8)	239 (46.5)	126 (81.8)	< 0.001
TBIL (> 17.1 μmol/L)	40 (4.0)	8 (2.5)	14 (2.7)	18 (11.7)	< 0.001
CREA (μmol/L)	69.0 [60.0, 80.0]	72.0 [63.0, 82.0]	69.0 [60.0, 80.3]	65.0 [57.5, 74.8]	< 0.001

*MVI* microvascular invasion; *AFP* α-fetoprotein; *PLR* platelet-to-lymphocyte ratio; *NLR* neutrophil-to-lymphocyte ratio; *ALT* alanine aminotransferase; *TBIL* total bilirubin; *CREA* creatinine

Patients in the high AAR group were more likely to have HBeAg-positivity (p < 0.001), positive HBV-DNA (p < 0.001), larger tumour size (p < 0.001), higher incidence of MVI (p < 0.001), and AFP > 400 ng/mL (p = 0.001) than those in the low AAR group. Patients in the high AAR group also had significantly higher PLR (p < 0.001) and higher NLR (p = 0.001) than those in the low AAR group. Regarding other liver function parameters, patients in the high AAR group had significantly higher ALT (> 40 IU/L, p < 0.001), higher TBIL (> 17.1 μmol/L, p < 0.001), and lower creatine level (p < 0.001) than those in the low AAR group. Generally, with a high AAR, the tumour was more advanced and systemic inflammation was higher.

### Relationship of AAR and hepatic inflammation and fibrosis

**Fig. 2:**
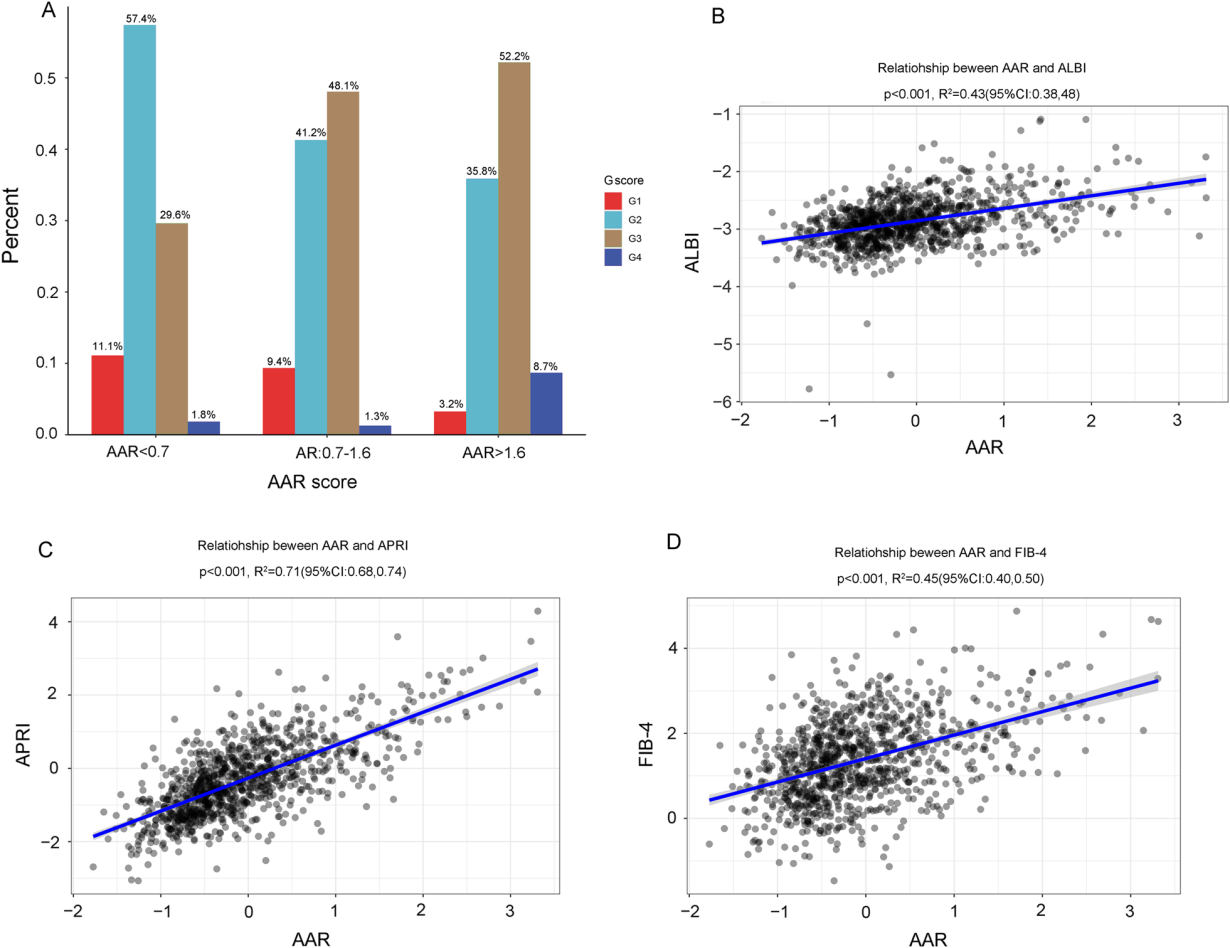
Correlation between AAR and hepatic inflammation and fibrosis score. **A** correlation analysis of AAR and the G score; **B**–**D** correlation analysis of AAR and the fibrosis score (ALBI, APRI, and FIB-4)

We further investigated the correlation between AAR and hepatic inflammation, cirrhosis, and fibrosis-related indices (ALBI, FIB-4, and ARPI; Fig. 2). Regarding the rate of patients with hepatic inflammation grade G1 or G2, there was a decreased tendency from the low-risk group to the high-risk group. In contrast, in terms of grade G3 or G4, there was an increased tendency from the low-risk group to the high-risk group. These findings suggest that AAR is positively correlated with hepatic inflammation. However, for liver cirrhosis, the correlation between both was not obvious. The ALBI, FIB-4, and ARPI were used to assess liver fibrosis. Despite the significant relationship between the risk score and ALBI or FIB-4, the correlation coefficient R^2^ was much small (0.36 and 0.45, respectively). Notably, the relationship between ARPI and the risk score was significant (R^2^ = 0.73, p < 0.001).

### RFS and OS stratified by AAR

**Fig. 3:**
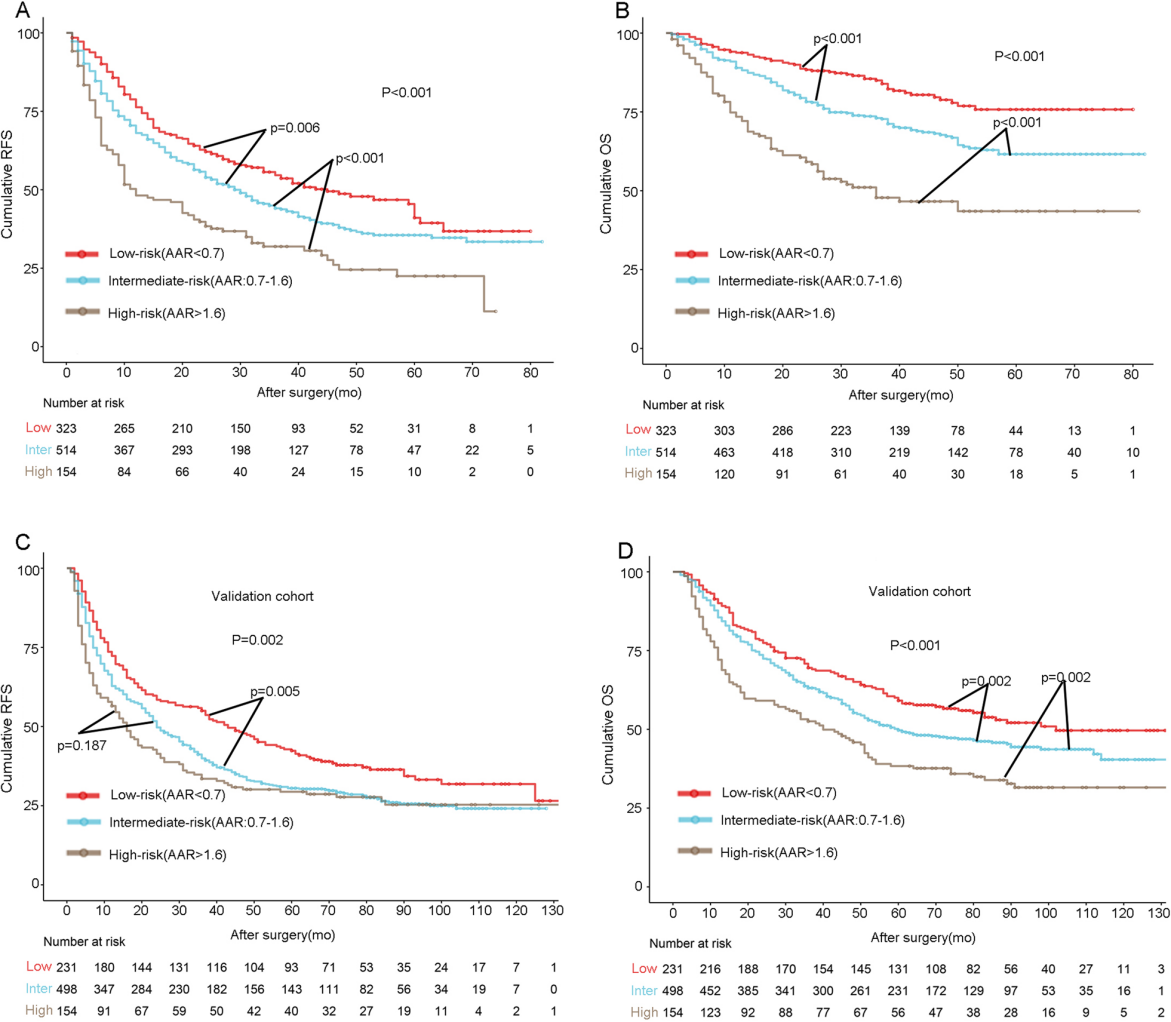
Kaplan–Meier estimates of survival by AAR in the primary and validation cohorts. **A** and **B** In the primary cohort, HCC patients in the high-risk group had worst prognosis in terms of RFS, whereas those in the low-risk group had best prognosis. A higher risk score was correlated with worse prognosis. **C** and **D** In the validation cohort, AAR (> 1.6, 0.7–1.6, and < 0.7) performed well in stratifying patients with distinguished prognosis. *RFS* recurrence-free survival, *OS* overall survival

Till the final follow-up, there were 295 deaths and 556 recurrences. Survival analysis was performed based on AAR. As expected, the score could significantly distinguish the OS of HCC patients between the subgroups (all: p < 0.001, intermediate-risk vs. low-risk: p < 0.001, high-risk vs. intermediate-risk: p < 0.001). In terms of the RFS, the risk score still performed well (all: p < 0.001, intermediate-risk vs. low-risk: p = 0.006, high-risk vs. intermediate-risk: p < 0.001). The 1-, 3-, and 5-year OS were 93.8%, 85.0%, and 75.8% for the low-risk group; 89.0%, 73.0%, and 61.6% for the intermediate-risk group; and 73.5%, 47.8%, and 43.5% for the high-risk group, respectively. The 1-, 3-, and 5-year RFS were 76.3%, 54.6%, and 41.1% for the low-risk group; 68.1%, 44.4%, and 35.5% for the intermediate-risk group; and 48.2%, 31.9%, and 22.5% for the high-risk group, respectively. With a higher score, the prognosis was worse (Fig. 3A and B).

**Fig. 4:**
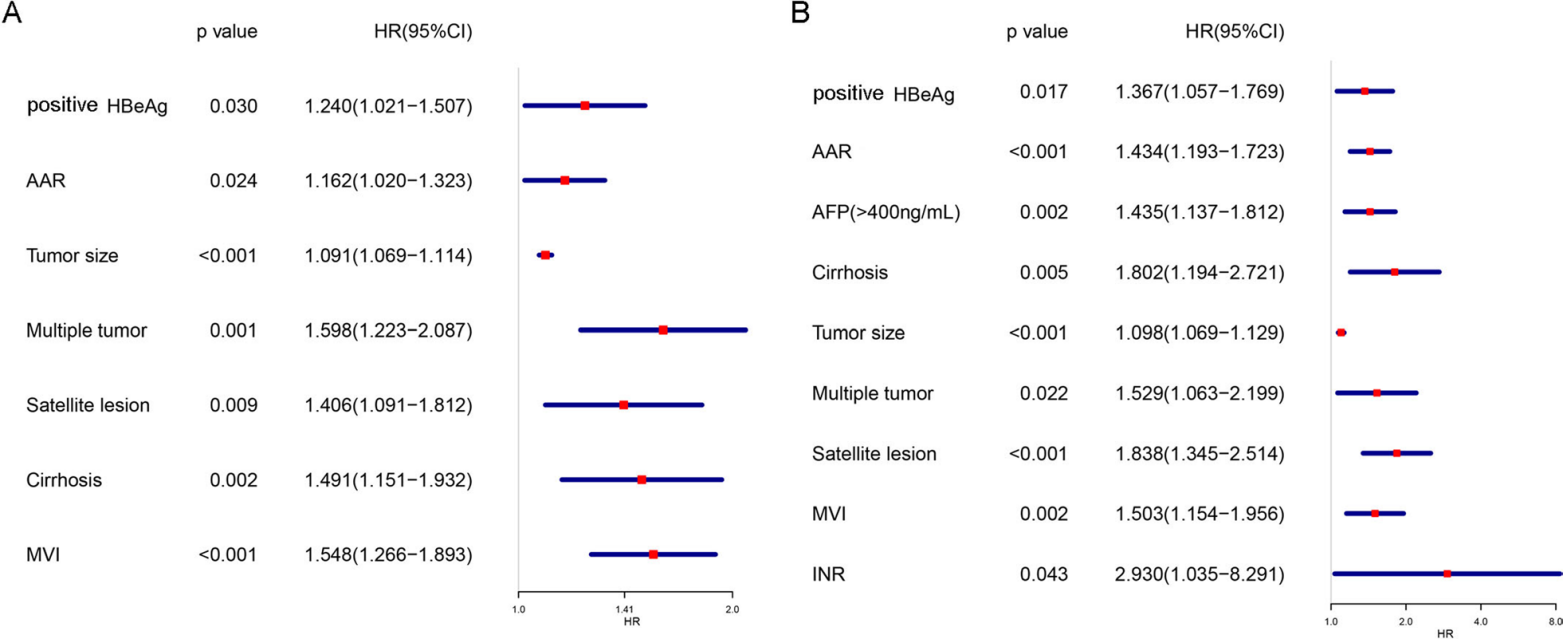
Multivariate Cox proportional hazards analyses of the clinicopathological factors associated with RFS and OS. **A** Factors associated with RFS in the multivariate analysis model. **B** Factors associated with OS. *RFS* recurrence-free survival, *OS* overall survival

As shown in Fig. 4, the multivariable analysis determined that AAR (HR 1.434, 95% CI 1.193–1.723; p < 0.001) was an independent predictor of OS in HCC patients undergoing hepatectomy, followed by tumour size, multiple tumours, AFP > 400 ng/mL, satellite lesions, MVI, liver cirrhosis, HBeAg-positivity, and INR (all p < 0.05). Similarly, the multivariable analysis determined that AAR (HR 1.162, 95% CI 1.020–1.323; p = 0.024) was an independent predictor of RFS in HCC patients undergoing hepatectomy, followed by tumour size, multiple tumours, satellite lesions, MVI, liver cirrhosis, and HBeAg-positivity (all p < 0.05).

### External validation

As shown in Additional file 1: Table S1, the clinicopathological features were distributed among the three groups similar to those in the primary cohort. A high AAR was associated with advanced tumours and active HBV replication. To validate the predictive role of AAR, we included another 883 patients who underwent hepatectomy between 2010 and 2014 at West China Hospital. AAR was constructed as abovementioned. As shown in Fig. 3C, and D, AAR could significantly distinguish the OS of HCC patients between the subgroups (all: p < 0.001, intermediate-risk vs. low-risk: p = 0.02, high-risk vs. intermediate-risk: p = 0.002). In terms of RFS, the risk score still performed well (all: p < 0.001, intermediate-risk vs. low-risk: p = 0.005, high-risk vs. intermediate-risk: p = 0.187) (Fig. 3C and D).

## Discussion

The present study is the first to evaluate the clinical significance of preoperative AAR in HCC patients undergoing hepatectomy. Using a multi-institutional database, we demonstrated that AAR was an independent risk factor associated with RFS and OS. The optimal cut-off values were 0.7 and 1.6, respectively. The low-risk (AAR < 0.7), intermediate-risk (AAR 0.7–1.6), and high-risk (AAR > 1.6) groups were constructed based on AAR. The high-risk group had the worst prognosis, whereas the low-risk group had the best prognosis. In the validation cohort, we found that the predictive ability of AAR in OS and RFS remained well.

HCC commonly occurred with the presence of an underlying liver disease, such as viral hepatitis and liver cirrhosis. Data from epidemiological survey revealed that HBV is attributed to 50% of HCC development worldwide. The liver function parameters such as ALT, AST, and ALB are commonly used to assess hepatic inflammation and preserved liver function. Hepatic inflammation indicated by elevated ALT levels increases the risk of HCC development, whereas ALT normalisation can reduce the risk of HCC development [15]. Moreover, serum liver chemistry alone or in combination can predict postoperative hepatic failure after hepatectomy [16]. Given the critical clinical significance of these indices, we proposed and demonstrated that combined liver function parameters (AAR) could predict the prognosis of HCC patients after hepatectomy. Patients with chronic hepatitis or HCC are more likely to have elevated AST levels. Moreover, Takeishi et al. reported that high AST level is significantly associated with tumour size and tumour capsule formation with cancer cell infiltration [17]. Serum AST levels might be associated with tumour malignancy in HCC patients. Serum ALB levels can reflect preserved hepatic function, nutrition status, and systemic inflammation. Based on the correlation analysis, ALB and AST had less influence on each other. Therefore, we combined the AST and ALB to form AAR. A high AAR commonly indicates high AST level and/or low ALB level. Compared with the low AAR group, patients in the high AAR group have more advanced tumours and higher serum TBIL and ALT levels than those in the low AAR group. Large tumour size and the presence of MVI are well-established risk factors associated with patients’ prognosis [18]. Interestingly, the mean tumour size was 4.0 cm in the AAR < 0.7 group, 5.0 cm in the AAR 0.7–1.6 group, and 8.05 cm in the AAR > 1.6 group. Consistently, the incidence of MVI was 13.3% in the AAR < 0.7 group, 25.9% in the AAR 0.7–1.6 group, and 33.1% in the AAR > 1.6 group. AAR appeared positively associated with the rate of MVI occurrence, which might aid to predict the status of MVI. Serum AFP level > 400 ng/mL predicts poor OS and RFS after hepatectomy in patients with HBV-associated HCC [19]. The rate of AFP level > 400 ng/mL was 31.9%, 38.3%, and 49.4% in the AAR < 0.7, AAR 0.7–1.6, and AAR > 1.6 groups, respectively. This evidence showed that AAR was associated with tumour aggressivity. In the present study, patients in the high AAR group had higher serum TBIL level. Elevated TBIL indicates a higher rate of postoperative mortality and long-term survival [20] and its combination with ALB has shown good predictive ability for HCC patients after surgery [6].

In addition to its relationship with tumour characteristics, AAR is related to inflammation. Hepatic inflammation plays a critical role in HCC development and progression [21]. Our study showed that the high AAR group had a higher incidence of severe histologic activity of G3 or G4, whereas the low AAR group had a higher incidence of mild histologic activity of G1 or G2. Thus, AAR could reflect hepatic inflammation to some degree. In our study, the majority of patients had a history of HBV infection, which was the main cause of hepatic inflammation. Interestingly, our result showed that the higher the AAR, the higher the rate of HBeAg-positivity and HBV-DNA > 10^3^ IU/mL. HBV replication could cause chronic liver injury and inflammatory cell infiltration. Consistently, serum ALT level was much higher in the high AAR group. Inflammatory microenvironment, in turn, contributes to tumour recurrence and metastasis [22]. HBeAg-positivity or high HBV-DNA load negatively impacts the prognosis of HBV-related HCC patients [23]. Therefore, AAR can effectively reflect the degree of liver-related inflammation. Inflammation-based scores, including the NLR and PLR, are associated with survival in patients with HCC [24, 25]. In the present study, NLR and PLR were associated with RFS and OS in the univariate analysis but were not independent predictors in the multivariate analysis. Because a high AAR was significantly associated with systemic inflammation, it could serve as an alternative for NLR and PLR. Hepatic and systemic inflammation were significantly higher in the high AAR group than in the low AAR group.

Liver fibrosis/cirrhosis is caused by chronic hepatic inflammation and is demonstrated to be an independent risk factor related to RFS and OS. However, in this study, no significant difference was noted in liver cirrhosis among the three groups. AAR could not reflect the status of cirrhosis. FIB-4 and APRI are scores for diagnosing liver fibrosis/cirrhosis. Interestingly, the correlation coefficient between AAR and APRI reached up to 0.73, whereas that between AAR and FIB-4 was 0.46. The APRI and FIB-4 are widely recommended for the evaluation of liver fibrosis; however, their performance remains controversial [26]. Therefore, the relationship between AAR and liver cirrhosis remains to be further elucidated. Based on the demographics, we found that AAR does not differ between female and male patients and no difference was noted in terms of age and the incidence of diabetes among the three groups. Multivariate analysis revealed multiple tumours, satellite lesions, HBeAg-positivity, and INR as independent risk factors related to prognosis. These variables were validated in various studies [11, 27]. In the present study, AAR was revealed to have good clinical utility in patients showing different clinical characteristics.

AAR was constructed based on preoperative liver function parameters (AST and ALB). The calculation process and risk stratification were relatively simple and could be easily applied. Notably, AAR could effectively reflect the tumour characteristics and hepatic inflammation as shown in the primary multi-institutional data or validation cohort. The association between a higher AAR and worse OS and RFS was also identified and maintained in the primary multi-institutional data and validation cohort.

There were some limitations to the present study. First, this was a retrospective study, which might lead to selection bias. To overcome this shortcoming, we included multi-center database to investigate the clinical significance of AAR. Additionally, we included a homogeneous group of HCC patients, which improved the external validity of the present findings. Second, the liver function test might be different in different hospitals. However, AAR might increase compatibility among the different hospitals when compared with AST or ALB alone. Finally, the liver function indices might be influenced by different antiviral drugs.

## Supplementary Information

**Additional file1: Table S1.** Baseline characteristics.

## Data Availability

The data that support the findings of this study are available from the corresponding author upon reasonable request.
