# Supplementary material for: Preoperative aspartate aminotransferase to albumin ratio correlates with tumor characteristics and predicts outcome of hepatocellular carcinoma patients after curative hepatectomy: a multicenter study
Source: BMC Surg. 2022 Aug 9;22:307. doi: 10.1186/s12893-022-01751-4 (PMC9364544; doi:10.1186/s12893-022-01751-4)
Supplement: Supplementary file 1 — Additional file1: Table S1. Baseline characteristics. [file 12893_2022_1751_MOESM1_ESM.docx]

Supplementary Table 1. Baseline characteristics

|  | all | Low-risk group | Intermediate-risk group | High-risk group | p value |
| --- | --- | --- | --- | --- | --- |
|  | 883 | 231 | 498 | 154 |  |
| Age (>60 y) | 190 (21.5) | 49 (21.2) | 112 (22.5) | 29 (18.8) | 0.622 |
| Gender (male, %) | 744 (84.3) | 185 (80.1) | 428 (85.9) | 131 (85.1) | 0.124 |
| HBeAg | 169 (19.1) | 32 (13.9) | 96 (19.3) | 41 (26.6) | 0.008 |
| HBVDNA (>10^3^IU/mL) | 424 (52.2) | 69 (31.7) | 256 (56.4) | 99 (70.2) | <0.001 |
| Cirrhosis | 565 (64.0) | 138 (59.7) | 327 (65.7) | 100 (64.9) | 0.290 |
| Tumor size (cm) | 5.0 [3.5, 8.0] | 4.0 [3.0, 5.9] | 5.4 [3.6, 8.0] | 8.0 [5.0, 11.8] | <0.001 |
| Tumor number |  |  |  |  | 0.892 |
| 1 | 766 (86.7) | 201 (87.0) | 430 (86.3) | 135 (87.7) |  |
| 2 | 90 (10.2) | 24 (10.4) | 53 (10.6) | 13 (8.4) |  |
| ≥3 | 27 (3.1) | 6 (2.6) | 15 (3.0) | 6 (3.9) |  |
| MVI | 278 (31.5) | 57 (24.7) | 149 (29.9) | 72 (46.8) | <0.001 |
| Satellite lesion | 123 (13.9) | 19 (8.2) | 77 (15.5) | 27 (17.5) | 0.012 |
| Branch of PVTT | 70 (7.9) | 10 (4.3) | 38 (7.6) | 22 (14.3) | 0.002 |
| Poorly tumor differentiation | 375 (42.5) | 93 (40.3) | 211 (42.4) | 71 (46.1) | 0.523 |
| BCLC staging |  |  |  |  | 0.013 |
| 0 | 56 (6.3) | 18 (7.8) | 32 (6.4) | 6 (3.9) |  |
| A | 673 (76.2) | 182 (78.8) | 380 (76.3) | 111 (72.1) |  |
| B | 83 (9.4) | 21 (9.1) | 48 (9.6) | 14 (9.1) |  |
| C | 71 (8.1) | 10 (4.3) | 38 (7.7) | 23 (14.9) |  |
| AFP (>400ng/mL) | 376 (42.6) | 81 (35.1) | 214 (43.0) | 81 (52.6) | 0.003 |
| PLR (>103.5) | 342 (38.7) | 90 (39.0) | 185 (37.1) | 67 (43.5) | 0.366 |
| NLR (>2.6) | 315 (35.7) | 78 (33.8) | 170 (34.1) | 67 (43.5) | 0.082 |
| ALT (>40IU/L) | 418 (47.3) | 30 (13.0) | 254 (51.0) | 134 (87.0) | <0.001 |
| TB (>17.1μmol/L) | 39 (4.4) | 8 (3.5) | 19 (3.8) | 12 (7.8) | 0.079 |
